# State of the art, recent advances, and challenges in the field of fungal mycelium materials: a snapshot of the 2021 Mini Meeting

**DOI:** 10.1186/s40694-021-00118-3

**Published:** 2021-11-10

**Authors:** Noam Attias, Achiya Livne, Tiffany Abitbol

**Affiliations:** 1 Materials and Surfaces, RISE Research Institutes of Sweden, Stockholm, 114 28 Sweden; 2 Department of Civil and Environmental Engineering, Ben Gurion University of the Negev, P.O.B. 653, Beer-Sheva, Israel

**Keywords:** Fungal mycelium materials, Biocomposites, Fungal materials, Hybrid materials, Sustainable materials

## Abstract

Material development based on fungal mycelium is a fast-rising field of study as researchers, industry, and society actively search for new sustainable materials to address contemporary material challenges. The compelling potential of fungal mycelium materials is currently being explored in relation to various applications, including construction, packaging, “meatless” meat, and leather-like textiles. Here, we highlight the discussions and outcomes from a recent 1-day conference on the topic of fungal mycelium materials (“Fungal Mycelium Materials Mini Meeting”), where a group of researchers from diverse academic disciplines met to discuss the current state of the art, their visions for the future of the material, and thoughts on the challenges surrounding widescale implementation.

## Background

Fungi are essential organisms in natural ecosystems, playing a vital role as decomposers and maintaining symbiotic relationships with organisms from other kingdoms. Fungi have been used by humans since ancient times, mainly for food processing, e.g., alcoholic beverages, baked goods, fermented cheese, vegetables, and beans [1]. Nowadays, new ways of engagement between humans and fungi are emerging, for instance in the development of structural materials based on the resilient network structure of fungal mycelium [2]. These materials can take the form of biocomposites comprised of a lignocellulose substrate bound into a cohesive structure by the hyphal mycelium network that forms the bulk surrounding matrix [3], or alternatively can be fully based on pure fungal mycelium [4–6]. Material homogeneity and properties are largely determined by the fungal species, growth conditions, the morphology and size of the substrate, and post-growth treatments, such as heating, pressing, and deacetylation or other chemical modifications [3, 4, 6–10]. The many variations available in the production phase lend to a versatile range of possible material outcomes and properties. Thus, fungal mycelium materials have enormous value as alternative materials for sustainable industry and in the transition to a circular economy and way of life [1, 11, 12].

**Fig. 1:**
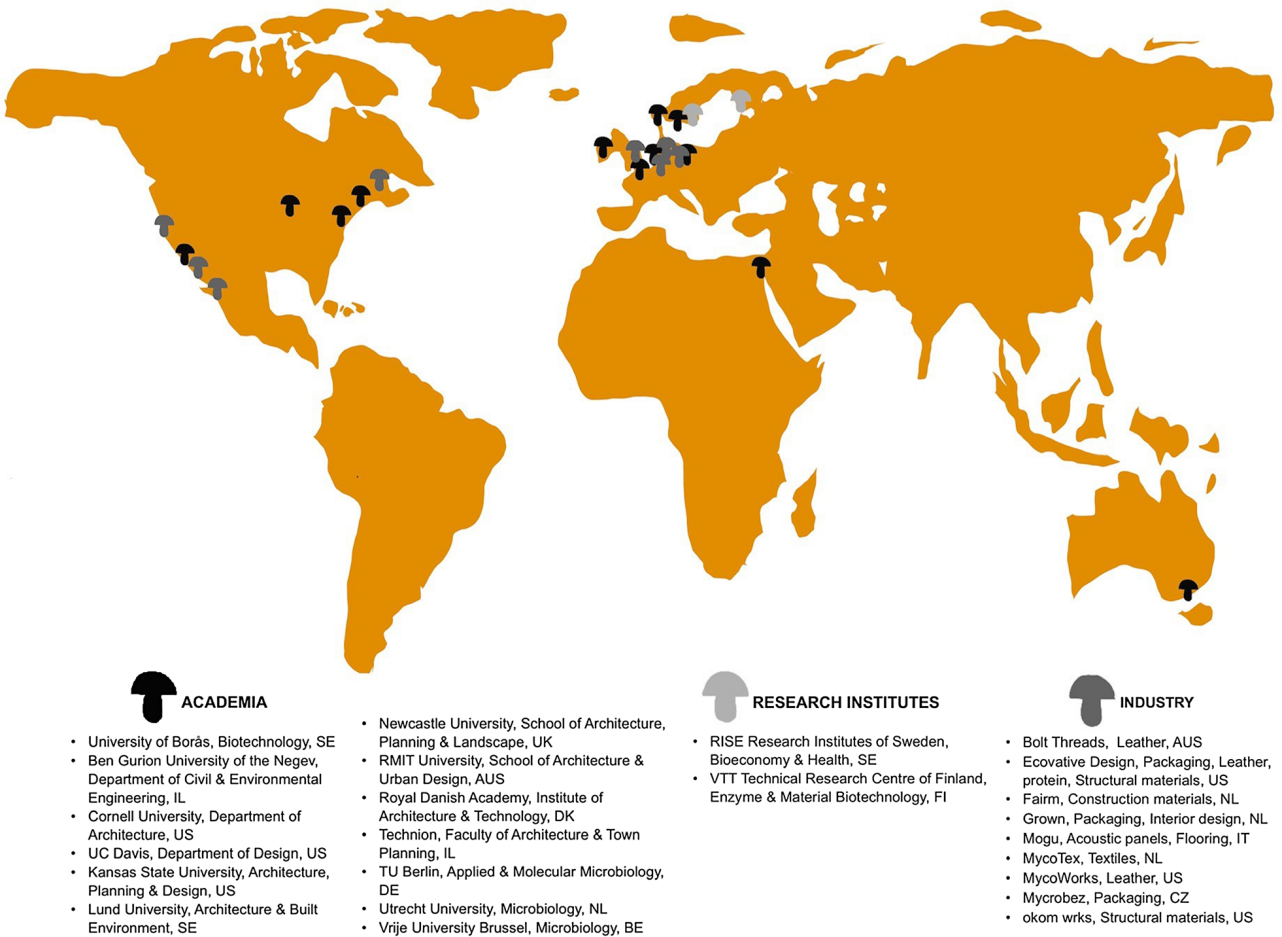
World map (and accompanying list) showing a geographic overview of some of the main research groups, institutes, and companies that are active in fungal mycelium materials research

As in any emerging field, comprehensive studies are needed to expand our current understanding of the material, particularly in relation to reproducibility, composition and homogeneity evaluation, substrate-fungal interactions, material processing, and the limits of the material properties that can be accessed from a given fungal-substrate pairing. At present, the fungal mycelium materials research community is comprised of researchers from distinct academic disciplines, including, materials science, biotechnology, engineering, architecture, art, and design, each with their unique perspectives and approaches to materials design and development. Figure 1 presents a (non-comprehensive) geographic overview of some of the main academic and industrial actors identified in the field at the time of writing. This figure captures the different groups that work with fungal materials and highlights the need for arenas that promote interactions between researchers that might not otherwise occur due to their separate disciplines and fields of study.

A general challenge that we identified in the available relevant literature relates to knowledge transfer gaps, either intentionally for reasons of intellectual property, or unintentionally through incomplete details provided in the materials and methods sections of scientific publications. This, however, destroys resources, leading to scenarios where the trials and errors of others are needlessly repeated. We think this aspect should be addressed through academic platforms that encourage knowledge sharing and dialogue between colleagues, for example dedicated conferences (such as the meeting described below), special issues in open access scientific journals (such as Fungal Biology and Biotechnology), and student exchanges between research groups.

To address these challenges, we hosted the “Fungal Mycelium Materials Mini Meeting” in August 2021, the first international meeting dedicated fully to fungal mycelium materials, where delegates from different research groups were invited to share insights into their work and recent data. The main goal of the day was to stimulate long-lasting and collaborative professional links between participants, supported by an informal meeting platform. As hosts, we promoted the open sharing of research ideas, expertise, and practical trouble-shooting tips by interspersing research presentations with brainstorming and discussion sessions. Due to the COVID-19 pandemic, the meeting was held online, yet still managed to achieve the friendly community atmosphere that was intended. Below, we summarize our perspectives of the main outcomes of the Mini Meeting, which we hope will become an annual event that continues to grow together with the research community.

## Synopsis of 2021 Fungal Mycelium Materials Mini Meeting

The meeting was co-organized by researchers from RISE Research Institutes of Sweden and Ben Gurion University of the Negev. As the main goal was to encourage open dialogue, setting the groundwork for future collaboration, we began the meeting with an icebreaker activity to initiate introductions. The remainder of the day was divided into two sessions of research talks, where each participant was given 25 min to present their work, answer questions, and solicit specific advice related to their field of study. In addition, a guest lecture on the topic of Life Cycle Assessment (LCA) was given by Tatjana Karpenja of RISE, who explained how LCA analyses can be used to determine the environmental footprint and overall sustainability of biobased materials, including those derived from fungal mycelium.

**Fig. 2:**
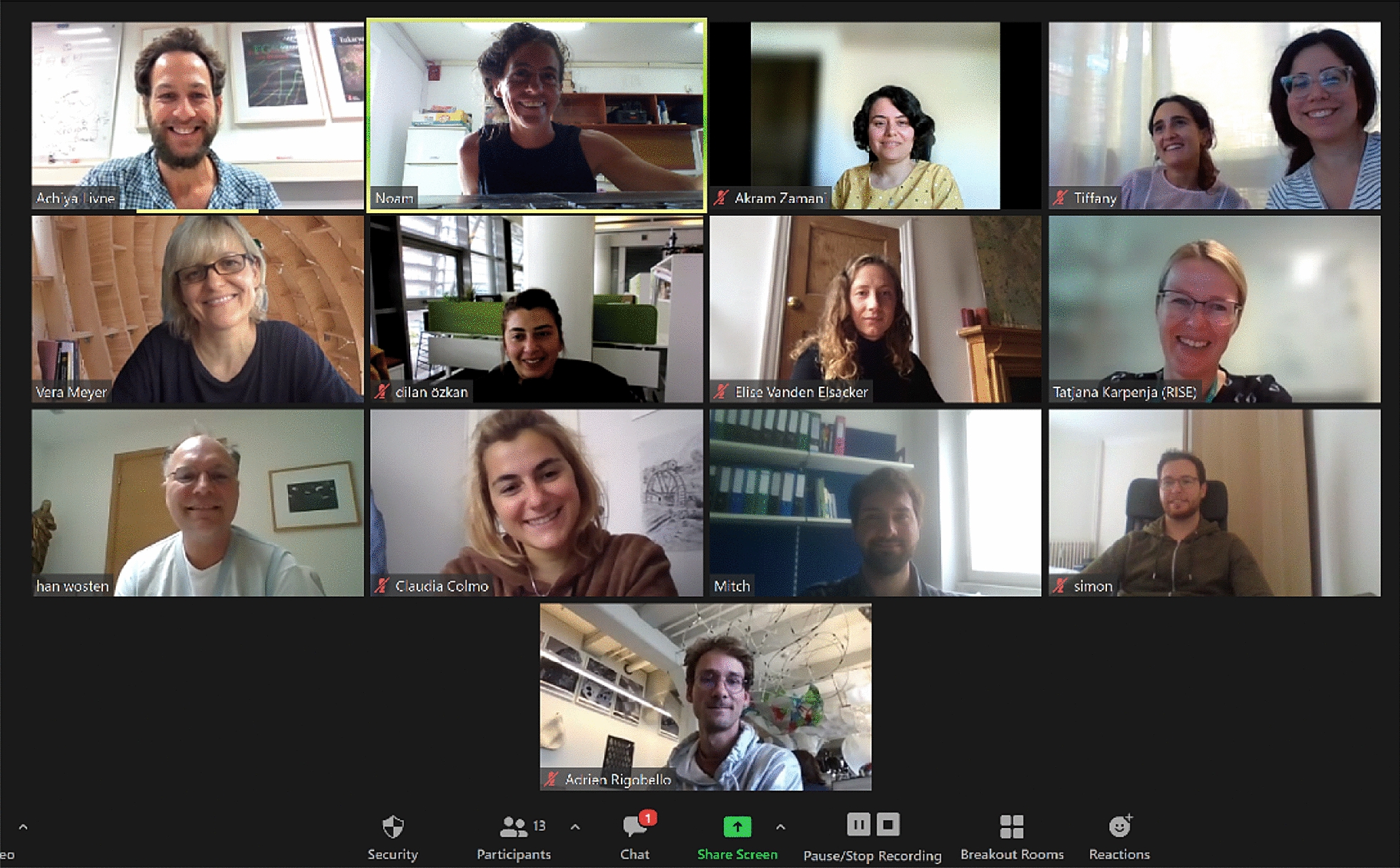
Meeting attendees, from left to right: Achiya Livne, Noam Attias, Akram Zamani, Maria Pita Guerreiro, Tiffany Abitbol, Vera Meyer, Dilan Ozkan, Elise Elsacker, Tatjana Karpenja, Han Wӧsten, Claudia Colmo, Mitchell Jones, Simon Vandelook, and Adrien Rigobello

The conference gathered 13 researchers from 10 research institutions with different fields and scientific backgrounds (Fig. 2): Achiya Livne (Department of Civil and Environmental Engineering at Ben Gurion University of the Negev), Noam Attias (Technion Israel Institute of Technology/RISE, Division of Bioeconomy and Health), Tiffany Abitbol (RISE, Division of Bioeconomy and Health), Han Wӧsten (Microbiology at Utrecht University), Vera Meyer (Applied and Molecular Microbiology at the Technical University of Berlin), Akram Zamani (Swedish Centre for Resource Recovery at the University of Borås), Elise Elsacker (School of Architecture, Planning and Landscape at Newcastle University), Simon Vandelook (Microbiology at Vrije University Brussel), Adrien Rigobello (Architecture and Technology at the Royal Danish Academy), Dilan Ozkan (School of Architecture, Planning and Landscape at Newcastle University), Claudia Colmo (Architecture and Technology at the Royal Danish Academy), Mitchell Jones (Materials Science and Technology Institute at TU Wien), and Maria Pita Guerreiro (Intern at RISE).

The talks emphasized the relevance of the field for a sustainable future through the promise of fungal mycelium materials as a substitute for common fossil-based materials or as new types of packaging, textiles, and construction materials. There was a consensus that further study and cross-discipline collaboration are needed to be able to truly harness the potential of fungal mycelium materials. Identified topics of mutual interest included LCA, scalability, material reproducibility, new processing strategies for resilient and functional materials, material aging, bioremediation, biodegradability, effects on the ecosystem, safety, durability, nomenclature (e.g., fungal mycelium materials/fungal materials and not “mycelium materials”, which are not necessarily derived from fungi), methodologies, material semiotics (socio-cultural association, physical, tactile, and aesthetic properties), regulatory aspects, commercialization potential, etc.

Additionally, the talks revealed fundamental gaps in the jargon used by the various disciplines in their approach to materials research, highlighting the need for common or translatable terminology. Evident throughout the meeting was the value of diverse perspectives as a tool to help push material exploration past the traditional boundaries of a given field of study, however the effectiveness of this approach relies on cross-disciplinary communication using mutually understandable terminology. All participants agreed that a dialogue between the design and scientific research approaches that currently populate this field (but that are usually independently applied) can be extremely valuable in the development of new and functional fungal materials. For instance, the tools of material semiotics used in material-oriented design research can complement scientific and engineering research approaches, and vice versa, scientific thinking and research can be used to elucidate structure-property-function relationships, which in turn can be used to inform the parameters of material design and application.

Until now, fungal mycelium materials research has been presented under the umbrella of conferences focused on other topics, such as sustainable materials in architecture and design, sustainable engineering, material science, general mycology, or fungal genetics, with limited presence of the broad fungal mycelium materials community. The Mini Meeting was meant to gather researchers active in all of these diverse fields, to build a community, and to support an open dialogue between the different disciplines – but this was just the start! We are currently on the lookout for additional opportunities to learn and interact. To this end, we are organizing future meetings, including a dedicated symposium hosted within the upcoming ACS Spring Meeting in San Diego (March 20-24, 2022). We are also looking forward to the upcoming 31st Fungal Genetics Conference in Asilomar (March 15-20, 2022). Both meetings are being held in California, with virtual attendance possible.

## Conclusions and outlook

The field of fungal materials is growing fast but has already started to divide into niche areas. The diversity of the field is a strength, but for this strength to be fully realized, a common language and open access to best practices for material production and characterization are needed. Opportunities to meet, like the Mini Meeting, can help to support collaborations and to expand the field of study by exposure to the trials, errors, and experiences of others in the field.

It is challenging to reach and bring together researchers across different disciplines. The Mini Meeting was a first step towards this direction, and we hope that it acts as the spark for future events, collaborations, and fruitful discussions.

## Data Availability

Not applicable.
